# The Prevalence of Natural Health Product Use in Patients with Acute Cardiovascular Disease

**DOI:** 10.1371/journal.pone.0019623

**Published:** 2011-05-09

**Authors:** Aws Alherbish, Theresa L. Charrois, Margaret L. Ackman, Ross T. Tsuyuki, Justin A. Ezekowitz

**Affiliations:** 1 Department of Medicine, University of Alberta, Edmonton, Alberta, Canada; 2 Division of Cardiology, University of Alberta, Edmonton, Alberta, Canada; 3 Pharmacy Services, Alberta Health Services, Edmonton, Alberta, Canada; Yale University School of Medicine, United States of America

## Abstract

**Background:**

Natural health products (NHP) use may have implications with respect to adverse effects, drug interactions and adherence yet the prevalence of NHP use by patients with acute cardiovascular disease and the best method to ascertain this information is unknown.

**Objective:**

To identify the best method to ascertain information on NHP, and the prevalence of use in a population with acute cardiovascular disease.

**Methods:**

Structured interviews were conducted with a convenience sample of consecutive patients admitted with acute cardiovascular disease to the University of Alberta Hospital during January 2009. NHP use was explored using structured and open-ended questions based on Health Canada's definition of NHP. The medical record was reviewed, and documentation of NHP use by physicians, nurses, and pharmacists, compared against the gold-standard structured interview.

**Results:**

88 patients were interviewed (mean age 62 years, standard deviation [SD 14]; 80% male; 41% admitted for acute coronary syndromes). Common co-morbidities included hypertension (59%), diabetes (26%) and renal impairment (19%). NHP use was common (78% of patients) and 75% of NHP users reported daily use. The category of NHP most commonly used was vitamins and minerals (73%) followed by herbal products (20%), traditional medicines including Chinese medicines (9%), homeopathic preparations (1%) and other products including amino acids, essential fatty acids and probiotics (35%). In a multivariable model, only older age was associated with increased NHP use (OR 1.5 per age decile [95%CI 1.03 to 2.2]). When compared to the interview, the highest rate of NHP documentation was the pharmacist history (41%). NHP were documented in 22% of patients by the physician and 19% by the nurse.

**Conclusions:**

NHP use is common in patients admitted with acute cardiovascular disease. However, health professionals do not commonly identify NHP as part of the medication profile despite its potential importance. Structured interview appears to be the best method to accurately identify patient use of NHP.

## Introduction

Complementary and alternative medicine (CAM) use has risen due to local, national and international availability of CAM practitioners, manufacturers and CAM products themselves [1]. Natural health products (NHP) are an important aspect of the CAM practitioner's practice and recently Health Canada's Natural Health Products Directorate (NHPD) defined NHP as vitamins and minerals, herbal remedies, homeopathic medicines, traditional medicines such as traditional Chinese medicines and other products including probiotics, amino acids and essential fatty acids [2]. Although variable definitions exist, CAM has been defined by the United States' National Institutes of Health National Center for Complementary and Alternative Medicine as “a group of diverse medical and health care systems, practices, and products that are not generally considered part of conventional medicine.” (http://nccam.nih.gov/health/whatiscam, accessed April 11, 2011) Whereas mainstream medications have often undergone rigorous evaluation and surveillance, NHP has traditionally relied upon patient or practitioner preferences, anecdotal evidence, often with limited data on clinical effectiveness or safety.

In the general population, CAM use had been the main focus of prior research, whereas NHP use has not previously been well defined and have been mixed with other supplemental and over-the-counter medicines. While CAM research has shed light on the emergence and increasing use of dietary supplements, herbal and homeopathic products [3], [4], and the incidence or prevalence of use by patients with cardiovascular disease is common [5]–[11], few high quality studies exploring NHP use have been done. Furthermore, estimates obtained via telephone or other survey methods may not capture detail necessary to draw conclusions on the nature of use, type of NHP used or accuracy of information obtained (for example, if patients do not consider vitamins to be NHP). The life-threatening nature of cardiovascular disease with its impact on life-style, the abundance of its pharmacologic therapy with increasing chance of drug-drug interactions and the coexistence of diverse co-morbidities make cardiovascular patients a special population where NHP use should be known.

Accordingly, we conducted a study describing the prevalence of NHP use in patients with acute cardiovascular disease in a large tertiary care hospital using direct structured interview. We also sought to determine the possible predictors of NHP use and the most reliable method of NHP in-hospital documentation by comparing the direct interview to other healthcare professional documentation.

## Methods

### Study Population

All patients with acute cardiovascular disease admitted to the University of Alberta Hospital between 7^th^ January to 6^th^ February 2009 were initially approached for participation in this study. To be eligible for the study, patients had to be older than 18, hospitalized for an acute cardiovascular condition, able to provide informed consent, read and speak English, and responsible for self-administration of medications. There were no other inclusion or exclusion criteria. If a patient was admitted more than once during the study period, the first admission was considered the index visit.

### Procedures and Data

The study protocol consisted of structured interviews inquiring about NHP use and pattern of use, followed by chart review for further data collection. All interviews were conducted by one interviewer (AA) to provide internal consistency on NHP generic names and categorization. The interviews were all carried out at the patient bedside setting. The interviewer received training, instructions and guidelines on NHP prior to the study by local NHP experts including a pharmacist with experience in NHP. This pharmacist accompanied the interviewer on random interviews (6% of interviews) for quality assurance.

The interview consisted of structured and open-ended questions about any NHP use, frequency of use, and patients were asked to provide the names of all NHP they used. This method has been previously used [5]. To facilitate the patient's understanding of the NHP, all patients were shown a pamphlet demonstrating pictures of the different NHP categories based on NHP definition by Health Canada's Natural Health Products Directorate (NHPD) in 2004 [2]. The frequency of use was divided into daily, weekly, monthly or seasonal and the patients were only considered an NHP user if they had used an NHP in the last year. If the patient provided a trade name, the interviewer used multiple websites to identify the product constituents.

After discharge, the medical record for each patient was examined to extract demographic and clinical data, and to chart NHP as documented in the pharmacist history, the admitting physician history, and the nurse's medication list. Each of these professions does a detailed intake medication history on admission for all patients.

### Statistical Analysis

The prevalence of NHP use in the study population is described using simple rates. The frequency of NHP intake among users, and the prevalence of each NHP category and product were further described. To assess the frequency of in-hospital NHP documentation by clinical staff, the frequency of NHP documentation by each profession (physician, pharmacist, nurse) was compared against the structured interview (considered the gold standard) using Kappa to quantify agreement. Previously defined classification of Kappa agreements were none (0), slight (0 to 0.2), fair (0.2 to 0.4), moderate (0.4 to 0.6) and substantial (0.6 to 0.8) [12]. McNemar's test was used to compare between the proportions of NHP documentation of the different professions using general NHP use as a dichotomous variable rather than unique products. A parsimonious multivariable model was constructed using univariate predictors from Table 1 (including baseline demographics, education, median income, medical history, medical therapy, and discharge diagnosis), which were entered into a logistic regression model if they had a p-value<0.20. The model was constructed using stepwise procedure, and results are presented using odds ratio (OR) and 95% confidence intervals (95%CI). Data was coordinated, quality assured, and analyzed by the Epidemiology Coordinating and Research (EPICORE) Centre at the University of Alberta. The health research ethics board at the University of Alberta approved the study.

**Table 1:** Baseline characteristics and discharge diagnosis.

		N (%)
Mean age, years (SD)		62 (14)
Male		70 (79.5)
Marital status	single	10 (11)
	married or common law	70 (80)
	separated, divorced or widowed	8 (9)
Highest Education	Less than high school	25 (28)
	High school	27 (31)
	College/technical school	23 (26)
	Undergraduate degree	2 (2)
	Postgraduate degree	11 (13)
Annual household income (Canadian dollars)	<20 000	3 (3)
	20 000–40 000	18 (21)
	40 000–60 000	12 (14)
	>60 000	19 (22)
	Not documented	36 (41)
Ethnicity	Caucasian	71 (81)
	Black	2 (2)
	Aboriginal	4 (5)
	Hispanic	2 (2)
	Asian	4 (5)
	Other	5 (7)
Discharge Diagnosis	Acute coronary syndromes	36 (41)
	STEMI	7 (8)
	NSTEMI	22 (25)
	Unstable angina	7 (8)
	Heart failure	18 (20)
	Ventricular arrhythmia	2 (2)
	Atrial arrhythmia	4 (5)
	Other cardiovascular disease	28 (32)
Comorbidities	Diabetes	23 (26)
	Hypertension	52 (59)
	Hyperlipidemia	44 (50)
	Smoking	25 (28)
	COPD/Asthma	9 (10)
	Musculoskeletal disorder	16 (18)
	GERD/PUD	10 (11)
	Renal impairment	17 (19)
	Hepatic impairment	1 (1)

Values are N (%) unless otherwise stated. STEMI = ST elevation myocardial infarction. NSTEMI = non-ST elevation myocardial infarction. COPD = chronic obstructive pulmonary disease. GERD = gastroesophageal reflux disease. PUD = peptic ulcer disease.

## Results

Of 107 patients identified, 11 were not in the target population (hospitalized for a non-cardiovascular condition, or unable to provide consent) and 8 refused to participate giving an overall response rate of 90%; the remaining 88 patients were enrolled and interviewed. Table 1 shows the demographic and clinical data of our patients. The mean age was 62 years (standard deviation [SD 14]) and 80% were male. The most common cause of admission was acute coronary syndrome (41%), and main co-morbidities included hypertension (59%), diabetes (26%) and renal impairment (19%).

### Prevalence and pattern of NHP use

The majority of patients used NHP (n = 69, 78%) and Table 2 reports the frequency of NHP use including the frequency of each NHP category and product used. Vitamins and minerals were the most commonly used NHP category (n = 64, 73%); other NHP categories frequently used included herbal products (n = 18, 20%), traditional Chinese medicines (n = 8, 9%), and others (probiotics, amino acids and essential fatty acids) (n = 31, 35%). With respect to single NHP use, multivitamins were the predominant NHP used (n = 41, 47%), followed by vitamin D (n = 34, 39%) and calcium supplements (n = 31, 35%). Other commonly used NHP included omega 3 fatty acids (n = 16, 18%), probiotics and garlic (n = 6, 7% each). Table 3 depicts the frequency of intake of NHP among those who used NHP; 75% reported daily intake while the rest had taken NHP less frequently. In the exploratory multivariable model to identify patient-level predictors of NHP use, only older age was associated with increased NHP use (OR 1.5 per age decile [95%CI 1.03 to 2.2]).

**Table 2:** Frequency of natural health product use.

		N (%)
Total NHP use		69 (78)
NHP use by category	vitamins and minerals	64 (73)
	herbal products	18 (20)
	homeopathy	1 (1)
	traditional medicines including Chinese medicine	8 (9)
	other (probiotics, amino acids, and essential fatty acids)	31 (35)
NHP use by specific product	Multivitamins	41 (47)
	Vitamin D	34 (39)
	Calcium	31 (35)
	Vitamin C	24 (27)
	Omega 3	16 (18)
	Vitamin B complex	11 (13)
	Magnesium	9 (10)
	Vitamin E	9 (10)
	Vitamin B12	6 (7)
	Probiotics	6 (7)
	Garlic	6 (7)
	Zinc	5 (6)
	Protein supplements	5 (6)
	Glucosamine	5 (6)

Other infrequently used products used included [4 uses each] coenzyme Q10, and flax oil; hawthorn, chondroitin, ginko biloba, ginseng, and vitamin B6; [2 uses each] serrapeptase enzyme, methylsulfonylmethane, cayenne pepper, peppermint, green tea, herbal tea, thai tea, folic acid, and replavite; [1 use each] lutein, white willow bark, motherwort, bilberry, lavender, eucalyptus, wintergreen, menthol, camphor, opti-i-see eye drops, bromelain, turmeric, Echinacea, graviola, rat root plant leaf, banana leaf extract, cranberry supplement, celery herbal, rosemary herbal, seaweed herbal, chamomile tea, carboxymethylcellulose, collagen supplements, vitamin B50 complex, gelatin, and agar.

**Table 3:** Frequency of NHP intake.

	N (%)
Daily	52 (75)
Weekly	7 (10)
Monthly	2 (3)
Seasonal	7 (10)
No response	1 (2)

### Frequency of in-hospital NHP documentation by clinical staff

When compared to the interview as the gold standard, the clinical history obtained by the admitting physician, pharmacist or nurse was generally poor (Table 4). The pharmacist history recorded NHP use in 41% (n = 28) of patients who reported NHP in the interview (Kappa 0.22, fair agreement), while physicians and nurses recorded NHP use in 22% (n = 15) (Kappa 0.1, slight agreement) and 19% (n = 13) (Kappa 0.08, slight agreement) of patients, respectively. There was a significant difference between the pharmacist history and the physician history (p = 0.019) and the nurse history (p = 0.002). Out of 275 unique NHP detected in the direct interviews, the pharmacist history identified 73 NHP (27%), the physician history documented 30 NHP (11%) and the nurse history recorded 33 NHP (12%).

**Table 4:** Frequency of natural health products documentation by clinical staff when compared to direct structured interview.

Profession	N* (%)	Kappa (degree of agreement)	N** (%)
Pharmacist	28 (41)	0.22 (fair)	73 (27)
Physician	15 (22)	0.1 (slight)	30 (11)
Nurse	13 (19)	0.08 (slight)	33 (12)

*N represents the number of matched histories of natural health product use (between profession and direct interview).

**N represents the number matched unique NHP products.

In order to explore reasons for non-documentation, charts were re-examined and a pharmacist history was identified in only (n = 45, 51%) of patients while the nursing and physician histories were available in all patients. When this was taken into account and only patients with a pharmacist history were examined and compared to direct interview, the pharmacist history recorded NHP use in (n = 28, 70%) of patients who reported NHP in the interview giving a kappa of 0.34 (fair agreement).

## Discussion

Our study demonstrates that NHP use is surprisingly common in patients with acute cardiovascular disease and that despite highly prevalent use, health professionals failed to recognize or record NHP as an important part of the medication profile. Pharmacists, who are expected to be closest to a gold standard since their training explicitly incorporates NHP, identified NHP in only 41% of all NHP users. The potential clinical impacts of missing this information are unknown, but would include drug interactions, adverse effects or other side-effects otherwise attributed to proven efficacious medications. Finally, as the use of direct structured interviewing was critical to identify use of NHP: future NHP and CAM studies should incorporate this methodology.

CAM use is popular in the general population globally; therefore we focused this study on NHP as it is more likely to cause side effects and drug-drug interactions with often limited supportive research-based evidence upon which to rely. In the general population, national telephone surveys of a representative population in the United states in 1990 (n = 2055 subjects) and 1997 (n = 1539 subjects) showed that the prevalence of herbal medicines increased from 2.5% to 12.1% and megavitamins from 2.4% to 5.5% [13]. A similar Canadian study of 12000 households found that 26% of a surveyed population had used herbal and/or homeopathic remedies in 1999, 41% in 2000 and 44% in 2002 [3]. This pattern and prevalence of use led Health Canada to clearly define NHP as a separate entity in 2004 [2], and using this NHP definition, a telephone survey in 2005 found that 71% of Canadians used NHP [14].

Studies of NHP and CAM have discordant results across populations with cardiovascular disease, geography and methodology utilized. In the United States, a subset of patients with cardiovascular disease derived from the 2002 National Health Interview survey showed the prevalence of herbal therapies and multivitamins to be 18% and 3%, respectively [13]. However, a direct mail out questionnaire to an outpatient cardiovascular population showed the prevalence of multivitamin to be 68%, vitamin E 6%, and vitamin C 13% [15]. A study that compared the use of nonprescription medications by patients with chronic heart failure showed 59% used vitamins and minerals and 38% used herbal and health food products [16]. However, in a developing country, the use of dietary supplements or herbs by Nigerian patients with hypertension was 96.6% [11]. Given this variability, standardized methodology should be developed and adopted for the purpose and population under study.

In previous NHP and CAM studies, the most consistent variable to correlate with a higher use was female gender [6], [13], [15], [17]. Other less consistent variables from the literature include younger age groups, higher level of education, and higher income [6]. In our study, older age was the only variable associated with higher NHP use in our study of patients with cardiovascular disease, who were generally older than those surveyed in the general population. However, given the increasing number of cardiovascular risk factors in younger patients, this may change in the future. Regardless, all patients should be approached regarding their use of NHP.

This study demonstrated poor documentation of NHP use by health professions despite prevalent use by patients and importance of these products. In order to assess possible drug-drug interactions, side-effect profiles, patient preferences, affordability and effect on disease outcomes, the use of NHP has to be identified as a first step. Failure to identify NHP as part of the medication profile may lead to continuation in administering costly and widely available NHP drugs without an evidence base to support their use in diseases with serious morbidity and mortality. Furthermore, some of the NHP identified included vitamin C and vitamin E, both tested in multiple adequately powered randomized trials and shown to have either no beneficial effect or potential harm [18]–[21]. Other NHP could result in serious adverse events such as bleeding from concurrent use of anti-platelets or anticoagulants with Ginko-biloba or garlic [22]. Similarly, proven efficacious medications may also be reduced by a NHP, for example, ginseng reduces the international normalized ratio (INR) when tested in a double-blind, placebo-controlled trial of patients taking coumadin [23]. Unlike pharmaceutical products approved for treatment of a specific disease and subject to careful scientific, public and governmental scrutiny, most of the NHP identified have little published high-quality evidence to support their widespread use. However, some NHP we identified have undergone extensive testing and are supported by international guidelines for disease such as osteoporosis (calcium and vitamin D) whereas others have been tested in a cardiovascular population and found to be either neutral (vitamin B complex) or potentially harmful (vitamin C and vitamin E). Even more concerning is the out-of-pocket expense to patients (equaling or exceeding that of many medications with a high grade recommendations from international guidelines). Finally, vulnerable patients are often susceptible to direct-to-consumer marketing that allows carefully worded claims of ‘cures’.

What is the reason for the identified gap in documentation? Previous literature had shown that many patients do not take initiative to inform health professionals about using NHP or CAM. Although one study reported that 80% of CAM users claimed that they had discussed CAM with their physicians,^7^ three other studies showed that over half of patients stated that their physician was unaware of their CAM use [24], [25]. When asked if it is important to consult a physician about NHP use, only 42% of patients completely agree and 29% somewhat agree on informing their physician [14]. Equally important and not previously rigorously studied, is whether or not health professionals in clinical practice are aware of the importance of including questions regarding NHP use in the history obtained at admission to hospital. Our study suggests that this is poorly documented and may be multifactorial.

The pharmacist history was chosen for further analysis because it was felt to be the closest clinical resource to the gold standard interview in identifying NHP use and because of the availability of pharmacists on the clinical team at our hospital. However, even this was less than optimal, in part because not all patients received a pharmacist medication history, but even when available, NHP were infrequently documented. This suggests that all three professions that would traditionally record medications on admission should improve NHP documentation, and education in both undergraduate and continuing education should emphasize the importance of this effort.

### Strengths and Limitations

The major strength of this study was the direct structured interview resulting in a smaller sample size. Direct interview for research purposes has implications for cost given the time involved, however, formal comparison of the cost-effectiveness of this technique versus telephone interview has not been done. However, comparing this method as a gold standard to the usual methods of medications documentation was a crucial objective of this study and thus has broader clinical research implications. Additional use in the clinical environment will enhance the utility of this method, as will enhancements in data portability between clinics, pharmacies, hospitals and patients. This study focused on duration, type and prevalence of NHP use, however, future studies should investigate the indication for each NHP use or to differentiate between prescribed or non-prescribed NHP and indications for each. We chose to categorize various NHP into groups, however, due to the nature of NHP categories and codification, inconsistency of labeling, and nomenclature made this challenging. NHP manufacturers should be encouraged to provide monographs similar to that of prescription-based pharmaceutical medications to aid clarification of the contents, dose, safety profile and indications as shown by published literature. This study explored the necessity of future larger studies using structured interviewing to investigate NHP use and to explore reasons of poor NHP recognition by medical profession in broader clinical practice.

### Conclusion

NHP use is common in patients admitted with acute cardiovascular disease. However, health professionals do not commonly identify NHP as part of the medication profile despite its potential importance. Structured interview appears to be the best method to accurately identify patients using NHP.
